# Improving access to geodetic imaging crustal deformation data using GeoGateway

**DOI:** 10.1007/s12145-020-00561-7

**Published:** 2021-01-18

**Authors:** Andrea Donnellan, Jay Parker, Michael Heflin, Margaret Glasscoe, Gregory Lyzenga, Marlon Pierce, Jun Wang, John Rundle, Lisa Grant Ludwig, Robert Granat, Megan Mirkhanian, Nathan Pulver

**Affiliations:** 1 Jet Propulsion Laboratory, California Institute of Technology, Pasadena, CA USA; 2 Indiana University, Bloomington, IN USA; 3 University of California, Davis, CA USA; 4 University of California, Irvine, CA USA; 5 City College of New York, New York, NY USA

**Keywords:** Science gateway, Web services, Geodesy, InSAR, UAVSAR, Earthquake, GeoGateway

## Abstract

GeoGateway (http://geo-gateway.org) is a web-based interface for analysis and modeling of geodetic imaging data and to support response to related disasters. Geodetic imaging data product currently supported by GeoGateway include Global Navigation Satellite System (GNSS) daily position time series and derived velocities and displacements and airborne Interferometric Synthetic Aperture Radar (InSAR) from NASA’s UAVSAR platform. GeoGateway allows users to layer data products in a web map interface and extract information from various tools. Extracted products can be downloaded for further analysis. GeoGateway includes overlays of California fault traces, seismicity from user selected search parameters, and user supplied map files. GeoGateway also provides earthquake nowcasts and hazard maps as well as products created for related response to natural disasters. A user guide is present in the GeoGateway interface. The GeoGateway development team is also growing the user base through workshops, webinars, and video tutorials. GeoGateway is used in the classroom and for research by experts and non-experts including by students.

## Introduction

Earthquakes can cause tremendous damage and loss of life yet a complete understanding of the processes that control them has been elusive. Traditionally geology and seismology have been used to study earthquakes, but over the last three decades geodetic methods have improved understanding of how strain accumulates in the crust and on fault systems, is released in earthquakes, and is transferred to other faults. Geodetic imaging uses various remote and in-situ observations to measure the detailed shape and deformation of the Earth, particularly at the surface, which can be analyzed and modeled to better understand the underlying processes. GeoGateway (https://geo-gateway.org) is a science gateway that provides tools for analysis, modeling, and response using geodetic imaging products in a web map-based interface. Users can rapidly and simultaneously visualize multiple types of data and download products or extractions of them for further offline analysis.

## Geophysical background and relevant data

Earthquakes happen nearly instantaneously, causing shaking; large events near populated areas can cause damage and loss of life. The stress accumulation that leads to earthquakes is a long-term process, driven by motion of Earth’s tectonic plates. Understanding crustal properties and how strain accumulates and is released is key to better understanding earthquakes and the hazards they pose. Seismometers measure earthquakes and their aftershocks, and geologic observations can document long-term fault motions, crack patterns, and offsets from surface rupturing earthquakes. Geodetic methods measure deformation from tectonic strain accumulation, earthquake fault slip, and postseismic response following earthquakes. Geodetic observations fill in a large data gap on temporal and spatial scales that are otherwise difficult to observe. The methods can also be applied to the study of landslides or other processes that deform or disrupt the land surface. GeoGateway focuses on measurement of crustal deformation from ground-based and remotely sensed geodetic imaging observations. The different types of measurements are sensitive to different temporal and spatial scales and can be fused to improve the overall measurement of surface deformation. Processes occurring at depth can then be inferred from the surface deformation.

**Fig. 1:**
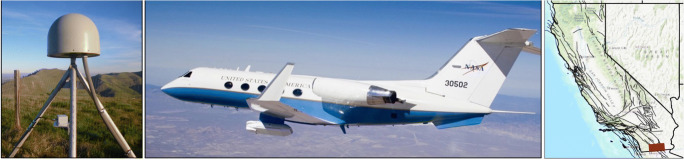
Instruments and data products. Left: GNSS station. Middle: UAVSAR pod is attached under the aircraft. The instrument looks left at an angle of 27–63° to the ground. Right: California showing faults in black and location of the displayed UAVSAR swath in Fig. 2 in red

Global Network Satellite System (GNSS), of which Global Positioning System (GPS) is a subset, produces position time series at distributed stations in a network (Fig. 1). Standard daily position time series can be used to measure long-term deformation, temporal changes, and station displacements from events such as earthquakes (e.g. Heflin et al. 2020). Accuracy of the products are 1–2 mm and <1 mm/yr velocity for horizontal displacement and velocity measurements respectively and about 3 mm and 0.5 mm/yr for vertical measurements (Heflin et al. 2020). Daily GNSS measurements are fully three dimensional and can provide continuous monitoring over decades but spatial sampling can be tens of km as is typically in California or hundreds of km in less tectonically active regions.

**Fig. 2:**
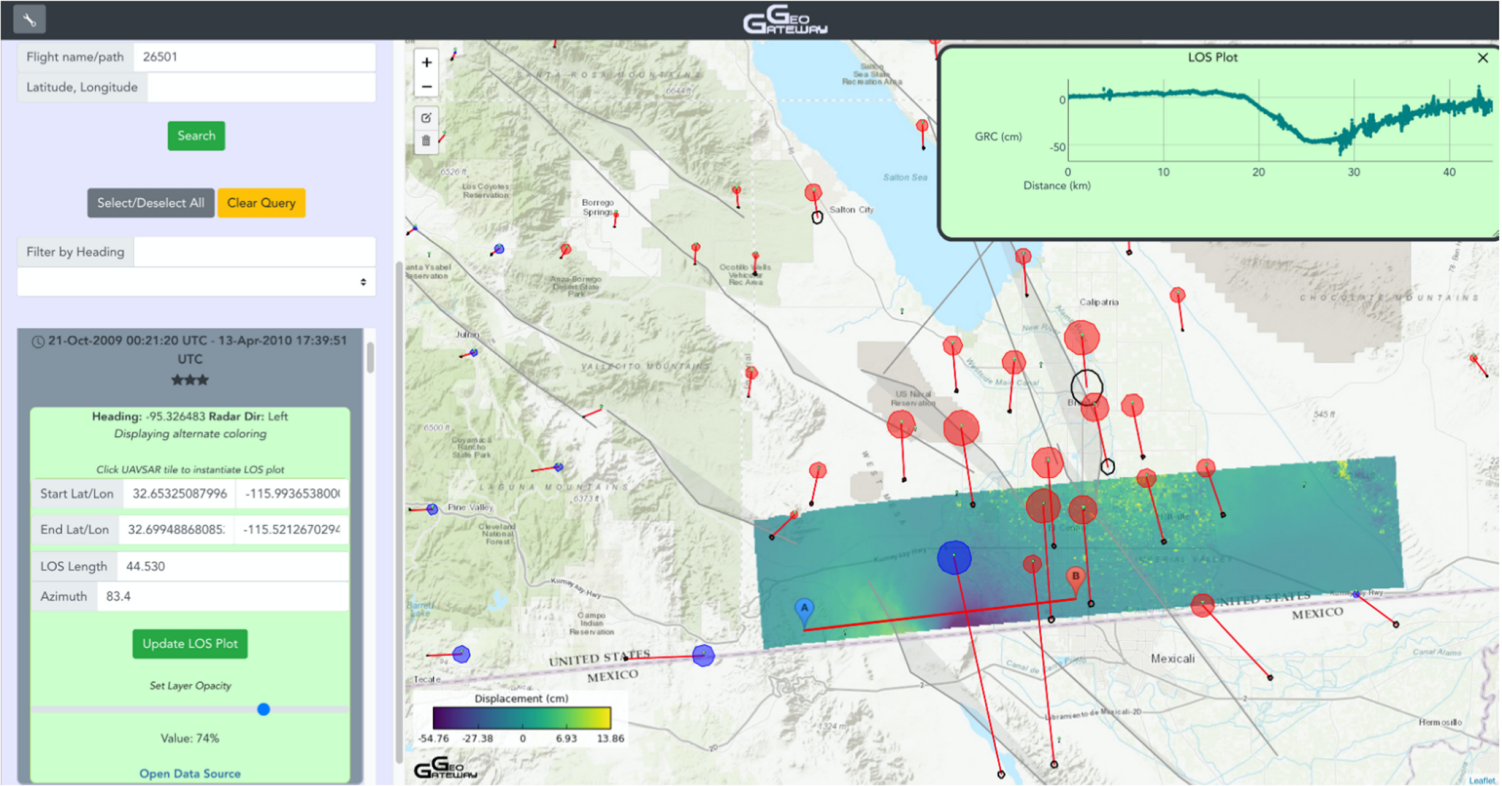
GeoGateway interface showing coseismic offsets from GNSS stations in red for the 2010 M7.2 El Mayor – Cucapah earthquake. Black error ellipses are at the arrowhead. Red or blue circles show uplift or subsidence respectively. The color image shows a UAVSAR product that spans the earthquake in time and covers the northernmost portion of the rupture. A line-of-sight displacement profile is extracted along the southeast portion of the product and results are plotted

Interferometric Synthetic Aperture Radar (InSAR) provides detailed images of range changes between pixels on the ground and the airborne or spaceborne instrument (Fig. 2). UAVSAR is NASA’s airborne InSAR platform, which is flown at an altitude of 12.5 km on piloted Gulfstream aircraft with precision autopilot (Hensley et al. 2008). InSAR uses pairs of images to produce line-of-sight changes between the ground and instrument resulting in detailed images highlighting concentrated and broad deformation patterns between the two image times. When the instrument repeats the same track on the first and second passes and the ground moves, the radar waves are offset, or interfere, between the two passes depending on how much the ground has moved. These phase changes that vary across the image appear as fringes in a radar interferogram. The range change can be converted to a displacement value, or unwrapped, from the known wavelength as long as the ground surface isn’t so disrupted that the radar waves decorrelate across the image. GeoGateway currently focuses on UAVSAR data, because products are produced operationally from this NASA platform. In the future the NASA ISRO Synthetic Aperture Radar (NISAR) mission, planned for launch in 2022, will also produce operational products. UAVSAR images only record line-of-sight changes in one direction but the images are spatially densely sampled with 1 m resolution for raw interferograms and 7 m for unwrapped products. Vertical and horizontal motions can be computed for observations collected from multiple directions.

Topography measurements support geophysical analysis and interpretation of other data. Geomorphology, the study of the physical features of the Earth’s surface and their relation to geology, relies heavily on measurement of surface topography. Topographic data are also important for interpreting radar data, particularly since the radar looks obliquely at the ground surface. Mountains or other steep terrain can block the radar signal, causing shadowing. Surface topography and associated imagery can be used to map locations of faults, measure offsets, such as streams offset by fault motion, from long-term geologic processes, and displacements from earthquakes on faults or due to slope failure and landslides.

Geologic earthquake fault observations are important to improve interpretation of the above data, for modeling, and to focus attention. Seismicity, or the size, location, depth, and type of earthquake, also helps with interpretation of the geodetic imaging data highlighting active areas and providing information on regional tectonics.

## GeoGateway

Science gateways (Lawrence et al. 2015) are science-centric web environments that help turn disparate data sources, analysis tools, computing resources, and scientific software into comprehensive end user environments that enable online research and support scientific collaboration. GeoGateway is an example of a science gateway that currently focuses on data access and interactive analysis of geophysical data sets. The main idea of science gateways is that they aggregate scientific software and data into online platforms through services with well-defined Application Programming Interfaces (APIs). These services may be operated by the gateway provider, or they may be operated by third party providers. Science gateways are responsible for organizing these general-purpose services into specific scientific usage scenarios through a combination of middleware and user interface components. GeoGateway (https://geo-gateway.org) provides tools for analysis, modeling, and response using geodetic imaging products, particularly those produced by NASA, in a web map-based interface. Users can rapidly visualize data and download products or extractions of them for further offline analysis.

### GeoGateway architecture and services

**Fig. 3:**
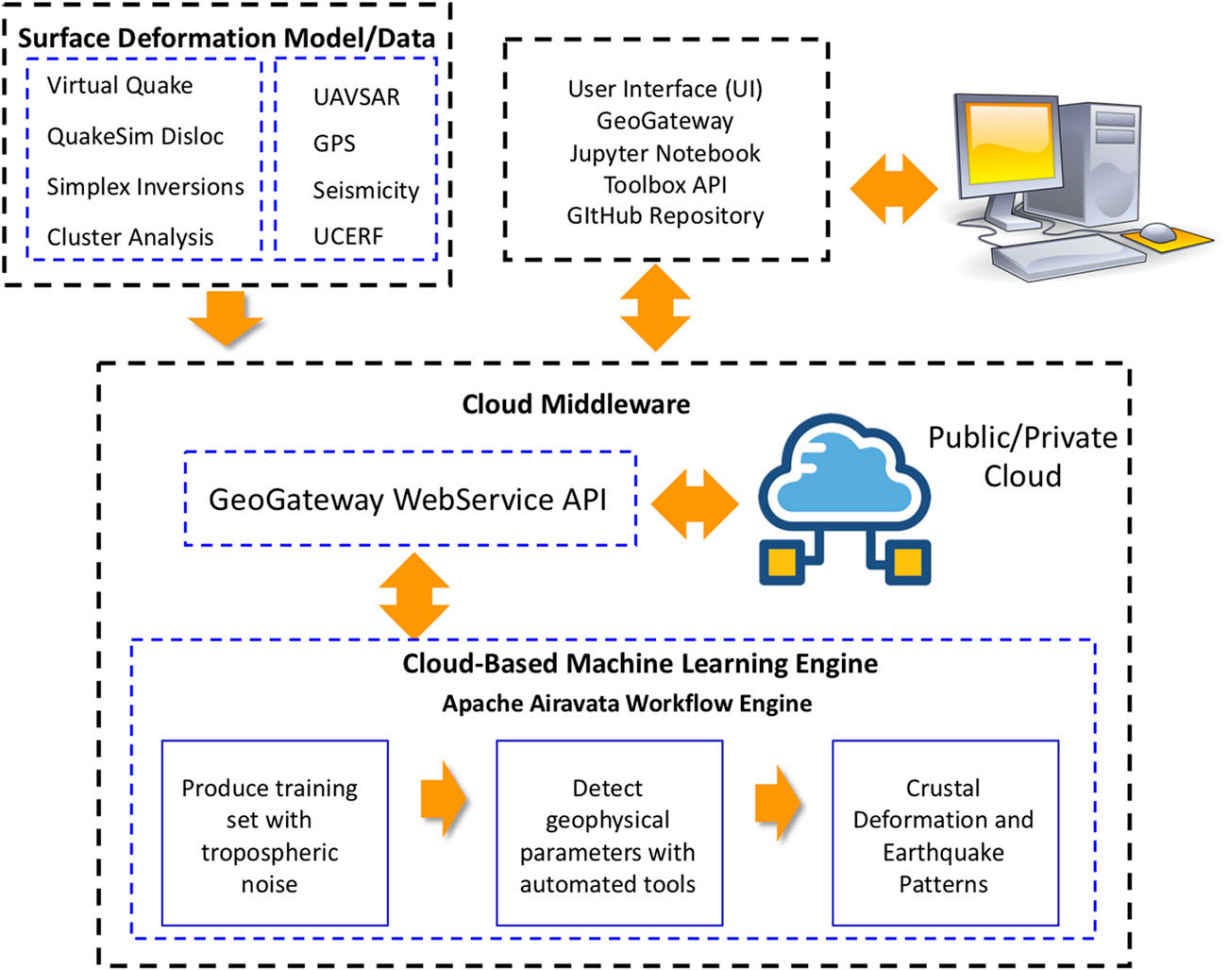
GeoGateway conceptual architecture. GeoGateway incorporates scientific applications and data into an online scientific platform using a service-oriented architecture with Web-based user interfaces

**Fig. 4:**
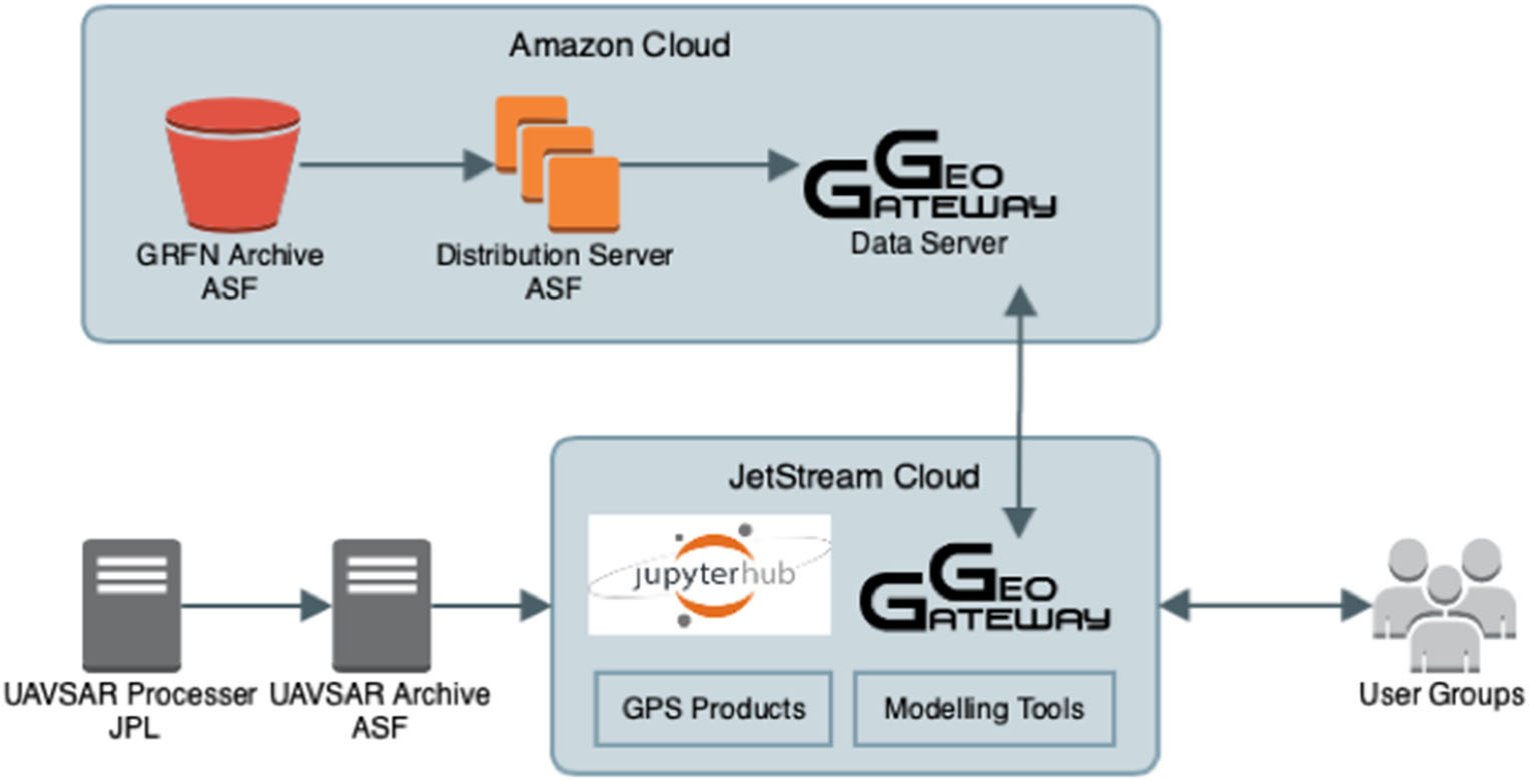
Architectural diagram showing UAVSAR and GRFN processing streams and other data and tools incorporated into GeoGateway ACF and served to the user community. Storage and preprocessing of GRFN products is in the AWS commercial cloud; GeoGateway ACF is built on the open source GIS platform and JetStream cloud (NSF XSEDE virtual compute resources), leveraging resources and reducing overall costs; Jupyter Notebooks is served as a collaboration tools among users

GeoGateway uses a service-oriented architecture with Web-based user interfaces (Fig. 3). GeoGateway provides core services for accessing UAVSAR, GNSS, and related data (Table 1). UAVSAR repeat pass interferometry (RPI) products (Hensley et al. 2005) and UCERF3 (Field et al. 2014) fault traces are provided by GIS services operated by the GeoGateway project and use the open source GeoServer software (http://geoserver.org/). GNSS data services (Heflin et al. 2020) are operated by NASA JPL (https://sideshow.jpl.nasa.gov/post/series.html), and seismicity data are obtained from USGS earthquake catalog. In addition, users can upload their own data sets in KML format (Fig. 4).

**Table 1:** Data products included in the GeoGateway core services. Most components allow for downloading of the extracted information for further analysis. RPI: Repeat Pass Interferometry. ASF: Alaska Satellite Facility. JPL: Jet Propulsion Laboratory. UCERF-3: Uniform California Earthquake Rupture Forecast 3 (Field et al. 2014). KML: Keyhole Markup Language. SCEC: Southern California Earthquake Center. We listed DOIs for products that have them. We also include the source web page or reference where applicable

Data	Description	Source
UAVSAR	Users can search for and select from over 1500 RPI products to display, recolor, stretch color, extract line of sight information (http://uavsar.jpl.nasa.gov; 10.5067/R0ARICRBAKYE)	ASF and JPL via local storage
GNSS	Tools to extract velocities, displacements, coseismic jumps, postseismic motions in specified region from the continuous global network daily positions (Heflin et al. 2020; 10.1029/2019EA000644; https://sideshow.jpl.nasa.gov/post/series.html)	JPL
Seismicity	Search function to display seismicity based on magnitude, location (https://earthquake.usgs.gov/earthquakes/map/)	USGS
Faults	UCERF3 fault traces are provided as a map layer, and can be displayed in different colors as overlay on top of other map products (Field et al. 2014; 10.3133/fs20153009)	SCEC
User Supplied	Users can upload their own custom KML files to layer on other map products.	User supplied
Response Products	Response products for the applications community from various natural disasters. Products currently include simplified RPI and radar polarimetry data for the Thomas Fire and Montecito Debris Flows (Donnellan et al. 2018a, b; 10.1029/2018EA000398) and stereo photogrammetry products for the 2019 Ridgecrest earthquake sequence (Donnellan et al. 2020; 10.5967/5sq2-rs60)	GeoGateway

GeoGateway has undergone a significant upgrade, involving a complete rewrite of its user interface, which we have maintained since 2014.The upgraded site was deployed at the end of 2020. We deprecated older Web framework approaches (AngularJS, Node.js, Google Maps) in favor of better supported, non-proprietary technologies (Vue.js, Django, Leaflet). We are also reviewing all software in order to provide better organization so that it will be easier to maintain and to modify by new developers. The new technology stack aligns with other projects in the Indiana University Cyberinfrastructure Integration Research Center, which develops and operates GeoGateway, increasing GeoGateway’s maintainability. The new technology stack is also aligned more closely with Apache Airavata middleware, which enables us to provide better support for executing scientific software asynchronously on managed, shared clusters and supercomputers. It also enables us to build on Apache Airavata’s security components, which can be used to provide improved group management and the ability to share results with collaborators in GeoGateway.

The collection of semi-autonomous services is complemented by execution services that can remotely execute relevant geophysical software. This software ranges in complexity from simple, interactive executions of Okada-based surface displacement calculations (Okada 1985) to the somewhat more computationally demanding inversion of these calculations to find best fit fault models, and to the intensive calculations needed to perform finite element-based simulations of fault systems.

Both data and execution services can be treated as relatively independent services and have APIs. Combining these services into useful workflows, such as linking the InSAR or GNSS data to one of GeoGateway’s geophysical applications, is the task of GeoGateway middleware. In order to manage the executions of software and transfers of data as input and output of analysis applications, GeoGateway provides an abstract container, called “Experiment” (Pierce et al. 2014), that collects all the basic information needed to conduct a specific user-driven workflow. This metadata collection is an immutable object in the system once the experiment is completed, but it can be cloned and used as the basis of other computations. It can also be shared with collaborators or made public.

GeoGateway middleware itself is accessed through its own set of APIs, which mediate calls between the user interface environment and the middleware. The user interface environment encapsulates common usage scenarios, which it translates into GeoGateway middleware calls. Many of the GeoGateway operations are interactive and map-based, so we make extensive use of client-side JavaScript libraries to create the user environment. The middleware thus also plays an important role in mediating service calls from users’ browsers to diverse remote services; the middleware acts as a broker for these calls, which otherwise would be subject to cross-site scripting restrictions on the client.

### Data available through GeoGateway

GeoGateway provides a collection of tools, APIs, and interfaces to access the data hosted on its GeoServer-based data servers (Table 1). These can be integrated with data from various data providers with Open Geospatial Consortium (OGC) standards (http://www.opengeospatial.org/).

UAVSAR RPI products are distributed as pure binary files with the metadata in a separated annotation file. A single file ranges in size from 500 Mb to 5Gb. To use the RPI data products in common GIS software such as GeoServer used in GeoGateway, ArcGIS, and QGIS, it is necessary to extract geo-spatial information from the annotation file to generate a proper header file. Neither the UAVSAR binary format or the Hierarchical Data Format version 5 (HDF5), specified for the upcoming NISAR mission, are an efficient file format to be used with GeoGateway’s online tools. GeoGateway and its predecessor QuakeSim project (Donnellan et al. 2006; Pierce et al. 2010; Parker et al. 2015) have converted 10 Tb UAVSAR RPI data products into GeoTiff format with the following steps: 1) Extract geo-spatial information from UAVSAR annotation file; 2) Convert UAVSAR from single band binary format to GeoTiff with the tiles and image pyramids options; 3) Calculate histogram and summary statistics; 4) Track metadata changes for the different processing procedures and parameters of UAVSAR images. 5) Register UAVSAR images and pre-rendered full-resolution image overviews with GeoServer. With these processing procedures, it enables GeoGateway to distribute the large UAVSAR image in full-resolution to the downstream applications through the Open Geospatial Consortium’s (OGC) Web Map Service Interface Standard (WMS) and Web Coverage Service (WCS) protocols. This allows rapid exploration and feature extraction of large collections of UAVSAR RPI datasets with open-source GIS software, enhances UAVSAR images with a dynamic coloring scheme (Wang et al. 2015), and provides a color blind-friendly coloring theme as an alternative visualization method to InSAR fringe patterns. For the NISAR mission, ASF will have on the fly converters to GeoTIFF, or better services for handling HDF5 will be available.

The advantages of adopting OGC standards for both data products and web services have been utilized by downstream projects, such as E-DECIDER (Emergency Data Enhanced Cyber-Infrastructure for Disaster Evaluation and Response; Glasscoe et al. 2014; Glasscoe et al. 2015), which employs remote sensing imagery, geodetic data, and tools from GeoGateway to automatically generate change detection products, critical infrastructure and deformation calculations triggered by an earthquake event.

### Rapid data exploration

An early motivation of support for overlays is the observation that UAVSAR data are complex and difficult to understand; historically a high level of technical competence has been required. Overlays of different data types in a Google Maps environment are illustrated in Fig. 2, which shows a UAVSAR product for the 2020 M7.2 El Mayor – Cucapah earthquake (Donnellan et al. 2014; Donnellan et al. 2018b), with line-of-sight displacement profile tool, and GNSS overlay. Note that while the tools are accessed by the tabs at the top left of the GeoGateway interface, any imaged item will be retained as the user moves to a new tab and tool to enable composite images. Users may toggle of any of the product overlays. Depending on the investigation, the user may visualize the following products in the map environment individually or in composite:

- Map Tools, including a capability to plot any KML/KMZ file onto the map, the simple UCERF3 fault traces, state and coastal boundaries, and the user’s current location, which is helpful in field work.
- UAVSAR, the interface for a global set of interferograms including the rapid line-of-sight profile tool, the dynamic coloring tool and the user rating tool.
- GNSS, for plotting a variety of displacement and velocity products.
- Seismicity, displaying recent earthquakes or else a user-specified subset of the USGS Advanced National Seismic System (ANSS) catalog.
- Forecasts, displaying experimental earthquake forecasting products.

We continue to add tools, including for modeling, but these are most often used for overlays. All are described in the User Guide. Using all would usually result in excessive clutter, but combinations can be highly helpful in initial exploration of physical processes behind the data sets.

In addition to the combination of UAVSAR and GNSS, overlays of fault traces, forecasts, and seismicity can provide insights about regional hazard. Overlaying fault traces, interferograms, and seismicity may enable discovery of fault slip on minor faults or segments of major faults.

Finally, combining the KML mapper with composite tool-based images allows overlay of GeoGateway scenes with user-supplied mapped items, such as location of strainmeters, field-surveyed locations, polygons marking areas of study, or third-party kmz files, subject to mapping limitations. Often a useful technique is to create markers, polygons, image overlays and so forth in Google Earth, collect them in a folder, and save the folder to KMZ for import into GeoGateway with the KML mapper.

### Modeling and simulations

GeoGateway currently supports forward modeling for an arbitrary number of faults using software called Disloc. Users can specify location, geometry and slip for each fault. In the current GeoGateway interface users upload an input file, which is then run on the backend. GeoGateway displays vectors and a simulated interferogram of the output. Users can specify the azimuth and elevation between the pixel on the map and the simulated instrument. A graphical user interface (GUI) that included map selection and parameter input was available in the earlier version thatran under QuakeSim. In the future we plan to implement Disloc using Jupyter Notebooks so that users will have similar GUI capability.

### Machine learning

GeoGateway hosts applications that apply machine learning algorithms to GNSS and seismicity data. GeoGateway’s machine learning tools are in transition. The GeoGateway team is developing numerous new machine learning and related applications, which we expect to integrate into the gateway as they mature. The interim integration that we will pursue in the next several months is to deploy JupyterHub and use it to make team-developed Jupyter notebooks available online. This will be integrated with GeoGateway to provide single sign-on, and team-developed notebooks will be developed to use GeoGateway’s underlying services via API calls.

One such approach uses a variant of hidden Markov modeling to search for anomalies in GNSS position time series data (Granat and Donnellan 2002; Granat 2004). We have supported RDAHMM classification of permanent GPS/GNSS stations since 2014 in the current GeoGateway; this application was also included in earlier versions of the gateway.The goal is to focus attention on subtle features in the data.

We are in the process of developing machine learning tools to further analyze the geodetic imaging products. We have developed unsupervised learning methods, which do not require labeled features, to identify GNSS vector clusters as well as to perform time series segmentation. Clustering GPS stations (Granat et al. submitted) not only has the potential for identifying useful scientific information, such as separating regions of post-seismic motion or ranking active fault systems, but also is a necessary initial step in other GPS analysis methods, such as those used to detect aseismic transient signals (Granat et al. 2013). Using this approach, desired features of interest can be selected, including some subset of the three displacement or velocity components, uncertainty estimates, the station location, and any other relevant information present in the data set. Based on those selections, the clustering procedure autonomously groups theGNSS stations according to a selected clustering method; some methods require that the number of groups be specified in advance, while others estimate the number of groups from the data. We have implemented this approach as a Python application, allowing us to draw upon the full range of open source clustering methods available in Python’s scikit-learn package (Pedregosa et al. 2011). The application returns theGNSS stations labeled by group in both tabular form and as a color coded KML file for overlay in Google Earth or GeoGateway.

The GeoGateway Earthquake Hazard Viewer under the nowcast tab provides several useful tools for the evaluation of earthquake hazard and risk. The forecast method is based on the Natural Time Weibull model developed by Holliday et al. (2016) and Rundle et al. (2016a, b). The Forecast Exceedance Probability Table on the web site shows the probability that an earthquake with magnitude exceeding M5, M6, M7, or M8 will occur within the selected region within the next one month, one year, or three years.

The Forecast Timeseries Chart shows the probability, as a function of time, that any point within a 50 km radius of any point in a selected region suffers an earthquake exceeding a given magnitude in the next year. The Global Forecast Map shows for any point on the map the probability that an earthquake of magnitude exceeding M6.5 occurs in the next year in a circle of radius 50 km of that point. The Ground Shaking Estimate is the Peak Ground Acceleration (PGA) that would be expected from an earthquake of given magnitude, location, and other earthquake variables.

An Open Hazards earthquake forecast is a real-time, seismicity-based forecast that computes probabilities in defined spatial areas using the Open Hazards Forecast Model. Open Hazards can compute a forecast at any time using real-time seismic catalogs to obtain the most current probabilities. The model is based on the Gutenberg-Richter magnitude-frequency distribution and on one of the most fundamental ideas in earthquake mechanics, the cycle of stress accumulation and release on major faults. Peak Ground Acceleration (PGA) is a measure of ground shaking. Open Hazards estimates PGA using a slightly modified form of the Cua-Heaton model (Cua and Heaton 2007).

Nowcasting is the prediction of the present, the very near future and the very recent past (Rundle et al. 2016a, b, 2018; Rundle et al. 2019). The term is a contraction of “now” and “forecasting” and has been used for many years in meteorology. Nowcasting uses proxy data to estimate the current dynamical state of a driven complex system such as earthquakes, neural networks, or the financial markets. Seismic nowcasting uses counts of small earthquakes as proxy data to estimate the current dynamical state of an earthquake fault system, or collection of interacting earthquake faults. The result is an earthquake potential score that characterizes the current state of progress of a defined geographic region through its nominal earthquake “cycle.”

The count of small earthquakes since the last large earthquake is the natural time that has elapsed since the last large earthquake. To use the nowcast calculator in GeoGateway , enter the latitude and longitude of a point on the map, as well as a name for the location. The system computes the nowcast within a 100 km radius circle for earthquakes of magnitude larger than 6.0.

## Developing a user community

Science gateways allow research communities to access shared data, software and services. The purpose of GeoGateway is to increase the value of existing geodetic imaging products to researchers, and allow users to efficiently find and use NASA geodetic imaging data products. GeoGateway bridges the gap between production and end-use of data products by simplifying the discovery of geodetic imaging products, enabling users to explore and integrate data products, and allowing researchers to easily share, publish and collaborate. Development efforts have focused primarily on implementation of technologies for facilitating data access and analysis by end users. Initial users and testers were members of the development team and their close associates. Publication of scientific papers (Donnellan et al. 2014, 2015,Donnellan et al. 2018a, b) showing GeoGateway applications and results led to interest by potential outside users. This has led to publications with other organizations such as the US Geological Survey (USGS; DeLong et al. 2016; Ponti et al. 2020), California Geological Survey (CGS; Dawson et al. submitted), and Geotechnical Extreme Events Reconnaissance Association (GEER; Brandenberg et al. 2020) and to work with various student research products. Informal surveys revealed technical challenges encountered by new users. To overcome this entry barrier, we developed tools and tutorials geared toward novice users. The target user communities include science users, hazard and resilience communities, and response organizations.

Geoscientists are the largest expected user group and the primary group targeted for early adoption. The target geoscience community includes geophysicists and geologists interested in active tectonics, crustal deformation, earthquakes, fault slip, and seismic hazard. Geoscientists start their careers as students; thus, initial efforts focus on cultivating new student users, and novice professional users. Science users, considered early adopters, have been introduced to GeoGateway via classroom instruction, online tutorials, a User Guide, presentations at scientific meetings, and workshops for hands-on training. From this training they can use GeoGateway tools for research projects or professional applications.

We have developed and implemented several strategies for increasing the GeoGateway user community. Initial efforts focused on scientific talks, posters and papers presenting research conducted in part with GeoGateway tools (Donnellan et al. 2014, 2015; Donnellan et al. 2018a, b; DeLong et al. 2016). Discussions with novice users and potential new users revealed technical challenges which created barriers to entry. To overcome this challenge, we developed tutorial exercises for use in undergraduate classes, and a GeoGateway User Guide for the website. The user guide is displayed by selecting the “Help” tab in GeoGateway. The tutorial exercises were tested in a class at California State Polytechnic University, Pomona in fall 2018, and the GeoGateway User Guide was rolled out during a half day workshop at the Seismological Society of America (SSA) Annual Meeting in April 2019 (Grant Ludwig et al. 2019). The classroom exercises and SSA workshop generated the largest single day user counts (41–42) to that date. Subsequently the Ridgecrest CA earthquake sequence generated the largest daily user count (55), suggesting that user training and development of a guide were effective in making GeoGateway more accessible to users following an earthquake. Future work will involve development of video tutorials to enhance the GeoGateway website, and offering of webinars for remote training.

In the 5-year period following April 1, 2015 GeoGateway has had more than 11,000 sessions and more than 600 repeat users. During the period4/1/19–4/1/20 over 340 repeat users visited the site and there were more than 2700 total sessions. Usage increased at times of significant events such as the 2019 Ridgecrest earthquake sequence or the 2018 Montecito debris flows. With documentation posted on the GeoGateway site, and team members giving tutorials at various scientific meetings (e.g. Grant Ludwig et al. 2019) and in upper level classrooms, we expect usage to continue to grow over time, particularly with the addition of these new tools.

## Science case studies

The ability of GeoGateway to present requested background information, geodetic information, earthquake catalog selections, and analysis products provides strong discovery and explanatory methods for a wide variety of cases of interest. Multiple tools produce products that through map-based overlay yield substantial insights into deformation processes. Here we focus on characterizing fault creep as identified in UAVSAR unwrapped interferograms, with comparison to seismicity in the time span corresponding to the interferogram pair of passes. Many other types of cases are supported, including GPS and UAVSAR indicators of regional deformation (Fig. 2), discovery and measurement of surface fractures triggered by an earthquake (Rymer et al. 2010; Parker et al. 2015), characterization of post-seismic processes, determining association of experimental earthquake forecasts with historic seismicity and deformation, estimation of fault frictional properties from UAVSAR interferogram sequences showing progressive triggered slip, to name a few (Donnellan et al. 2018b).

### Fault creep: San Andreas and superstition hills

Creeping faults are faults that have evident slip apart from the occurrence of local earthquakes, even small earthquakes. Examples well-covered by UAVSAR observations in the time span 2009-present include the roughly constant slip found on the central San Andreas Fault (SAF; Liu et al. 2011), the intermittent slip on the Superstition Hills Fault (SHF) and also parts of the SAF in the Salton Trough, and parts of the Hayward Fault and Rodgers Creek Fault east of the San Francisco Bay. We demonstrate the utility of GeoGateway tools for two case studies, focusing on the central SAF and the SHF.

**Fig. 5:**
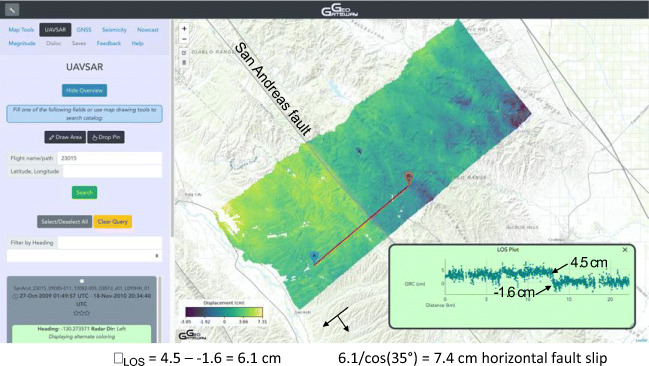
The creeping section of the San Andreas fault can be seen as a color discontinuity in a UAVSAR interferogram. Gray lines indicate UCERF 3 fault traces. The San Andreas fault is labeled. Note UCERF 3 represents fault segments as straight lines, so the actual fault trace varies somewhat. Line 23015 is shown between King City and Coalinga in the timeframe 27 October 2009–18 November 2010. A line of sight (LOS) profile show 6.1 cm of LOS slip across the fault.. The aircraft is flying to the southwest looking left. The elevation to the airborne instrument is higher near the aircraft (~63°) and lower on the far end of the swath (~27°). An elevation angle of 35° using the assumption that all of the motion is horizontal yields 5 cm of horizontal fault slip in the 13-month timeframe

Fault creep is observed in UAVSAR RPI products near the central part of the creeping SAF segment (Fig. 5). This case study and the next use three GeoGateway tools, accessed by the tabs at upper left of the interface. Note that mapped items are retained as we move from tab to tab, creating a composite mapped image of all the items created. UCERF 3 fault lines are displayed by checking the corresponding box in Map Tools, one of the tabs at top left in GeoGateway. The colorful interferogram is selected and displayed by selecting the UAVSAR tab, then selection of a flight line and repeat pass interferogram are made using methods illustrated in the GeoGateway user guide. Similarly, the line-of-sight (LOS) profile is created on a user-selected line as shown in the user guide. Geometry for converting line of sight to horizontal or vertical displacements is shown in Donnellan et al. (2014). The profile values may be saved to a file for further analysis. The projected displacement across the fault is shown to be 2.2 cm. Assuming pure strike-slip motion, the non-projected actual slip scales this by 1/cos(*D*)and 1/cos(*E*), where *D* is the difference of the fault strike and the radar look azimuth (140–138) = 2^o^, and *E* is the elevation angle = 38^o^. So the actual right-lateral slip is 2.2/cos(2^o^)/cos(38^o^) = 2.8 cm in 12.7 months. The Seismicity tab enables selection of earthquakes from the ANSS catalog, according to constraints of a geographical coordinate box, a time span, and magnitude limits, as described in the user guide. The resulting set of seismic events are displayed as red dots, sized according to magnitude.

**Fig. 6:**
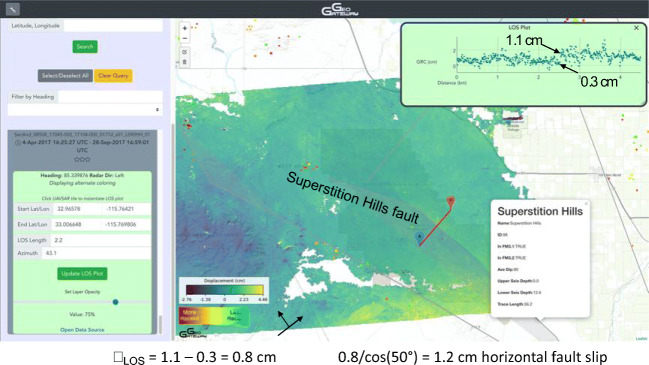
Creep on the Superstition Hills fault can be seen in a 5-month UAVSAR span. Gray lines indicate UCERF 3 fault traces. The Superstition Hills fault is labeled. Pop-up window that is displayed when a user selects a fault is also shown. Line 08508,immediately SW of Salton Sea, is shown for the time period 4 April 2017–28 September 2017. Seismicity > M0 is shown as dots for the matching time period: April 4, 2017 to September 28, 2017 (ANSS catalog). This profile shows slip projected on the Superstition Hills Fault is 1.2 cm over 6 months, far from any seismicity

Similar results of the same procedure are found for line 08518 (Fig. 6), selecting a profile crossing the Superstition Hills Fault for an interferogram in 2017.The profile indicates projected slip at this point on the fault is 2.2 cm over nearly 6 months, 10 km distant from any seismicity. In this case the difference of the fault strike and the radar look azimuth = 2^o^, and the elevation angle = 38^o^, indicating right-lateral motion = 2.7 cm. In both cases over 2.5 cm of right-lateral strike slip motion, assuming no dip-slip fault motion, is deduced where the profile crosses the fault. The closest seismic event in the radar pass time span is over 10 km away and about M 3. Therefore, these are creeping events, not caused by local earthquakes.

This Superstition Hills Fault creep event is found here in an interferogram based on a radar pass time span that includes the M 8.2 Chiapas earthquake of September 8, 2017 (UTC date) located in southern Mexico, suggesting that in this case slip may be triggered slip from a 2800 km-distant large earthquake. Triggered creep from this earthquake was detected on the southernn San Andreas fault (Tymofyeyeva et al. 2018). Plausibility is enhanced by a collection of ground water steps and spikes across the continental United States coincident with the Chiapas event, documented at https://waterdata.usgs.gov/blog/earthquake.

### Landslides

**Fig. 7:**
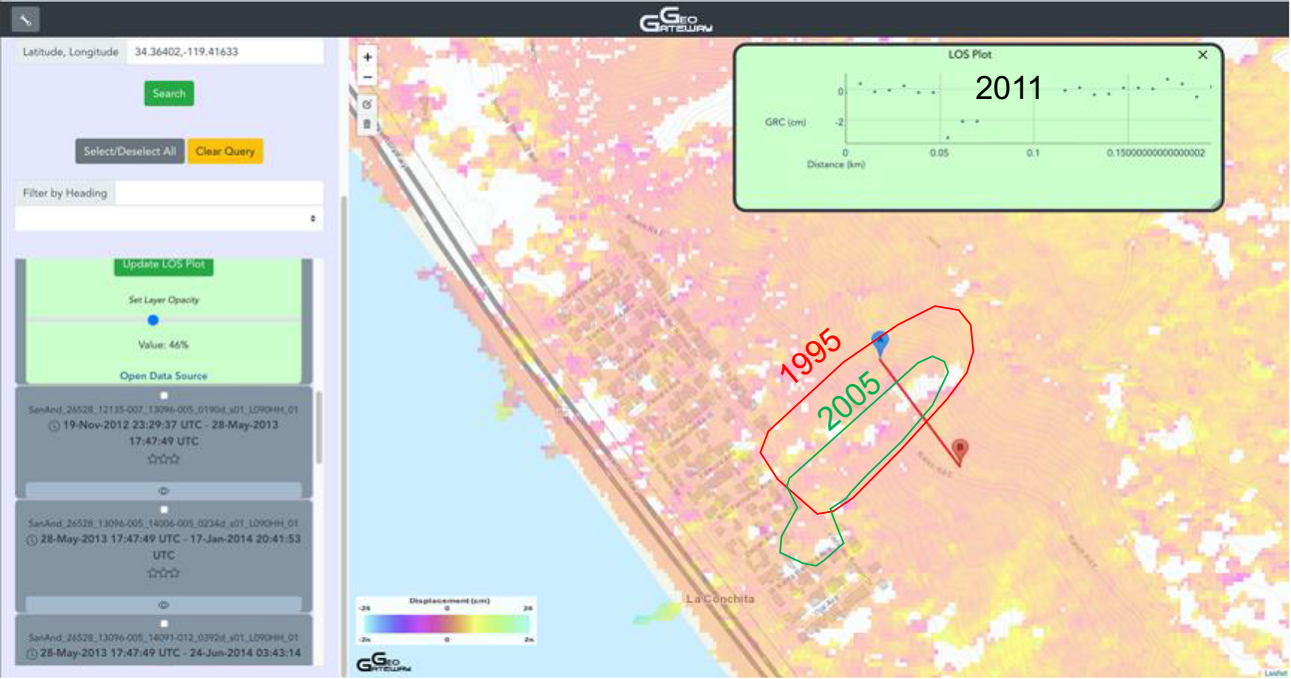
Region of the La Conchita Landslides between Ventura and Santa Barbara, California. 1995 and 2005 landslide outlines are shown in red and green respectively from Jibson (2005). Line of sight profile across landslide shows differential motion or decorrelation (loss of data coherence between passes) across the landslide

GeoGateway was used in a study to determine the ability to identify landslides in UAVSAR image pairs. The study focused on the La Conchita landslides of 1995 and 2006, which were catastrophic to the local community (Pulver et al. in preparation). The 1995 slide destroyed or damaged a total of 14 houses over the course of two events that were six days apart. The slide in 2005 was considerably smaller, but caused much more destruction and loss of life. In 2005, the mass movement destroyed or severely damaged 36 houses, and killed 10 people. GeoGateway provided an easy way for early exploratory analysis within a web browser as well as a download link for further exploration. The profile tool was also used in determining ground range change across the La Conchita landslides (Pulver et al. in preparation). The KML mapper feature proved useful when drawing these profiles. It allowed the input of a KML with both the 2005 and 1995 slide areas defined as polygons, and displayed them on the map. The aim of the study was to find an identifiable feature of the landslide in the UAVSAR image to be able to spot slides like this before they happened. The La Conchita slide was clearly visible in the data, even in images collected many years after the sliding event (Fig. 7). The difficult aspect of this is identifying areas of sliding without previous field knowledge of their existence. In this study, the profiles pulled directly from geo-gateway were analyzed for patterns and anomalies in an attempt to identify a signature of the known unstable region of La Conchita.

## Disaster response

GeoGateway has facilitated research for non-specialists in geodetic imaging that has resulted in publications as well as event response (e.g. documented in Donnellan et al. 2018a). Critical lifelines such as water, power and gas are vulnerable to failure under tectonic deformation, including fault rupture and distributed deformation. GeoGateway tools and data can provide important insight and analysis of spatial and temporal distribution of deformation to identify and prioritize strengthening or repair of vulnerable infrastructure. GeoGateway could also be used in response to earthquakes to identify areas which are likely to have distributed damage. Examples of potential end users include lifeline utilities and state or local offices of emergency services (OES). Utilities typically retain geoscience and engineering consultants who could effectively utilize GeoGateway for analysis of infrastructure vulnerabilities. The geoscience consultants are a subset of the user community identified as the most important potential user group.

GeoGateway has helped to support response efforts in events such as the South Napa (DeLong et al. 2016) and La Habra earthquakes (Donnellan et al. 2015). UAVSAR images were used to ground truth deformation that occurred as a result of the earthquakes. Partners at the California Geological Survey and US Geological Survey have found the tools useful in interpreting where damage and deformation has occurred as well as its magnitude. For example, they were able to identify areas of damage at the Napa County Airport after the M6.0 South Napa earthquake (Ponti et al. 2019) and surface fractures from the 2020 El Mayor – Cucapah earthquake (Rymer et al. 2010).

Partners in the response community have used GeoGateway in several events and the products have proven useful in identifying extent of damage and deformation. GeoGateway has partnered with the California Geological Survey, the US Geological Survey, and the California Earthquake Clearinghouse. In addition to providing products to active responses, GeoGateway has partnered with the Clearinghouse to provide products for their many earthquake exercises. This has provided the opportunity for the response community to become familiar with the tools between events in a non-emergency setting. This has also broadened the user base to partners such as CalOES, CalEPA, city and county jurisdictions, as well as the California National Guard.

As participants in California Earthquake Clearinghouse exercises, the goal was to help create both common operational data as well as a workflow in order to aid in the efficient delivery of products to end users. As a partner in this work, GeoGateway worked to provide geo-enabled data products to the various partners of the Clearinghouse. Thus, in the case of actual event response, the project was poised to deliver products quickly into the hands of partners for decision making. We learned that often the quickest method to get these into their hands was via email, though they later could ingest the data into their geospatial tools via our web services. When working in the field, large data transfers are often not possible, so smaller files are more convenient, then larger files are accessible once responders return to their field offices.

In the case of response, GeoGateway provided rapid modeling information, showing where deformation may have occurred to steer collection of UAVSAR data. This aided end-user partners with interpreting where deformation and potential damage may have occurred from an earthquake. Geospatially enabled data, which is available via web services from GeoGateway, is a preferred mechanism for delivery for the response community. GeoGateway will continue to evolve to accommodate the end user community, as new tools become available. The project strives to best serve the community by responding to their needs through technical interchanges and continued involvement in exercises and event responses.

### GeoGateway future directions

**Fig. 8:**
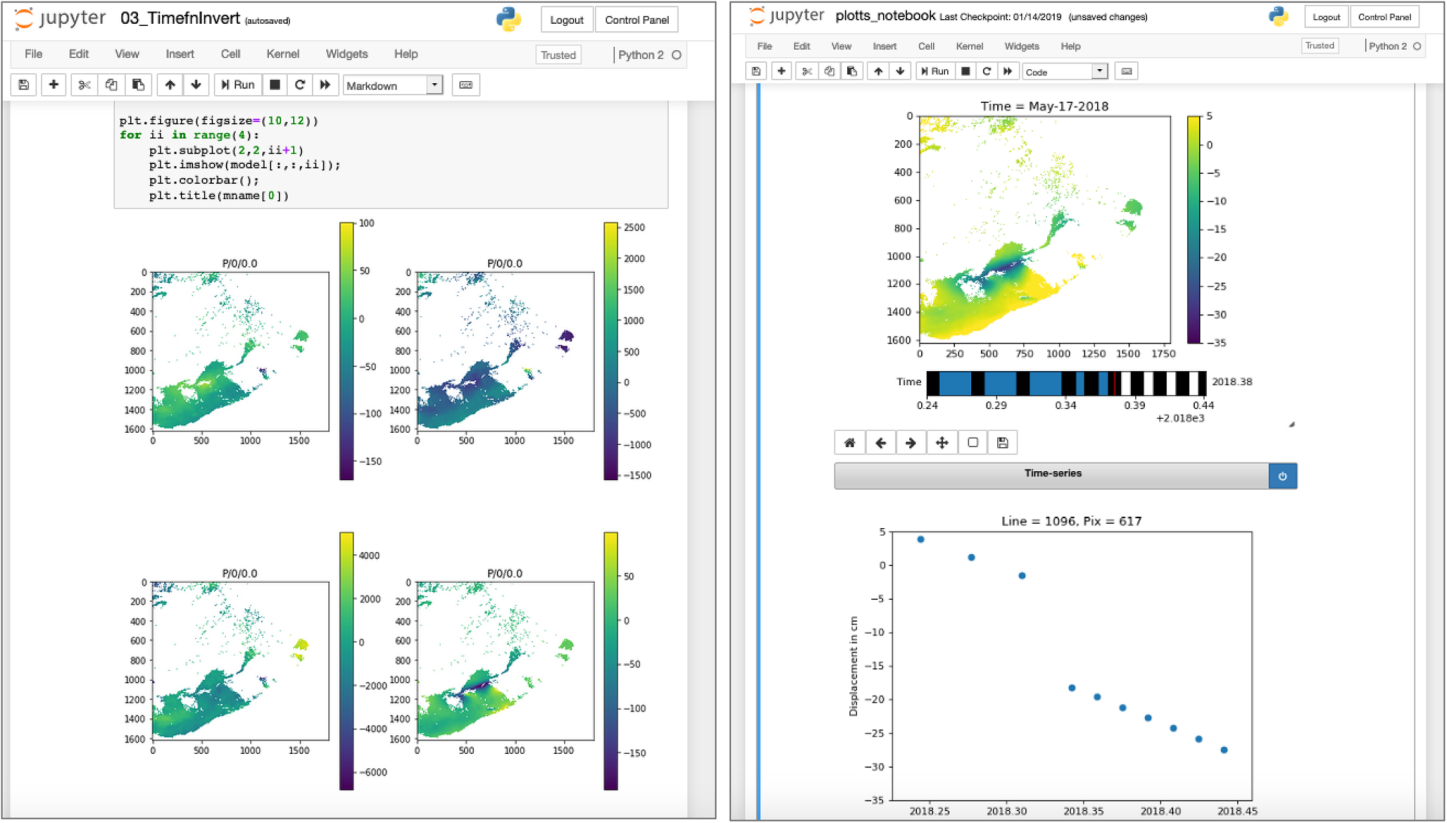
Processing the sample Getting Ready for NISAR (GRFN) data products with GIAnT in Jupyter notebook

GeoGateway’s architecture is designed to keep end user environments and abstract middleware tasks separate, allowing us in principle to replace the user environment with other approaches. This has become increasingly important with the emergence of Jupyter Notebooks and their online hosting via JupyterHubs. GeoGateway’s user interface layer can be replaced with online Jupyter Notebooks running in Jupyter Hubs (Fig. 8); this approach provides a simpler operational approach to users running notebooks locally. Notebooks provide a flexible alternative to the fixed user interfaces of the Web-based GeoGateway client environment. With sufficient documentation through example notebooks, advanced users can create their own custom notebooks that are specific to their scientific research efforts. Code can be hidden to provide less experienced users with a more intuitive interface. These notebooks can be shared via GeoGateway’s GitHub repository. Notebooks are also a powerful way to prototype new user interface capabilities. The default GeoGateway Web interface represents a set of tools and capabilities that we consider to be commonly useful. Creating usable interfaces by distributed development teams with diverse expertise is challenging using traditional methods. Notebook interfaces will allow our core team to more quickly prototype new interfaces and enhancements to existing interfaces before pushing these into production usage.

We are working on improved application support for GeoGateway. GeoGateway has simple support for scientific applications, which we are enhancing to support a greater range of applications of interest to our team and the geophysical community. We are working on implementing simpler ways of adding and configuring new applications, both from within our team and from third-party providers. These applications need to run on diverse resources, such as supercomputers, campus resources, and computational clouds for full analysis of complex and large volume geodetic imaging data. The Apache Airavata software for science gateways (Marru et al. 2011) provides us with a much more sophisticated mechanism for executing scientific workflows, managing user identities and permissions, integrating with diverse resources, and managing data transfers.

Finally, we focus on the development of tools that can assist with the more demanding computational requirements of uncertainty quantification and on the development of middleware that can provide better support for the development and execution of machine learning-based applications. Machine-learning applications have several needs. Currently GeoGateway hosts GNSS analysis and seismic analysis machine learning tools. The first searches for anomalies in the GNSS time series data (Granat and Donnellan 2002; Granat 2004). We also display earthquake forecasts and users can create nowcasts (Rundle and Donnellan 2020). We are creating new machine learning applications, which we must verify and validate. GeoGateway then needs to execute mature validated applications as software as a service. This builds on services already part of the Apache Airavata middleware, although there are opportunities to improve support for containerization of codes.

As part of future GeoGateway developments we are developing clustering algorithms to divide GNSS velocity and displacement fields across discontinuities, generally associated with fault boundaries (Granat et al. submitted). We are improving the seismic nowcasting methods. We also plan to display modeling and simulation products related to earthquake and tsunami simulations and improve the automated machine learning tools. Tsunami simulations will involve a new developmental code called Tsunami Squares. This code is a cellular automaton code to propagate ocean bottom disturbances across basin-wide distances. An advantage of this method is that dry-land tsunami run-ups can be easily calculated. The tsunami software must run offline due to the complexity, but we plan to display products from these codes on the GeoGateway website. Future developments also include the deployment of tools to explore geodetic image and cluster velocities or displacements. This helps identify boundaries of deformation and provides a means to rank fault activity. Because geodetic products are non-uniform both in space and time, we are developing approaches to interpolate the data, based on the machine learning algorithms to provide uniform pixel by pixel time dependent displacements that can be used for interpretation and ingestion into geophysical deformation process models. Ultimately our goal is to increase the user base by providing tools to simplify and promote rapid analysis of geodetic data products and move toward expansion of GeoGateway to include user contributed applications.
